# Incidence and Risk Factors of Gestational Diabetes Mellitus: A Prospective Cohort Study in Qingdao, China

**DOI:** 10.3389/fendo.2020.00636

**Published:** 2020-09-11

**Authors:** Guoju Li, Tao Wei, Wei Ni, Ai Zhang, Jun Zhang, Yuhan Xing, Quansheng Xing

**Affiliations:** ^1^Qingdao Women and Children's Hospital, Qingdao University, Qingdao City, China; ^2^Qingdao Women and Children's Health Care and Family Planning Service Center, Qingdao City, China; ^3^Department of Paediatrics, Faculty of Medicine, The Chinese University of Hong Kong, Hong Kong, China

**Keywords:** gestational diabetes mellitus, body mass index, gestational age, risk factors, Qingdao

## Abstract

**Background:** Obesity and maternal age are the two most important factors independently affecting the risk of gestational diabetes mellitus (GDM). However, the age differences in the association between obesity and GDM remain unclear. The objectives of this cohort study included: (1) to determine the current incidence of GDM in Qingdao; and (2) to evaluate the risk factors for GDM, such as the interaction between pre-pregnancy body mass index (BMI) and age.

**Methods:** The cohort included 17,145 pregnant women who registered at 15 to 20 gestational weeks from August 1, 2018, to March 1, 2019. A 75-g 2-h oral glucose tolerance test (OGTT) was conducted for each participant at 24–28 gestational weeks. The age-adjusted incidence of GDM was calculated using logistic regression. Multivariate logistic regression analysis was used to identify risk factors. Interaction between age (reference group <30 years) and BMI (reference group <25 kg/m^2^) was determined using strata-specific analysis.

**Results:** The incidence and age-adjusted incidence of GDM in Qingdao were 17.42 and 17.45%, respectively. The incidence of GDM appeared to increase steadily with age in all pre-pregnancy BMI groups (all *P* < 0.05). Older age (≥30 years), gestational BMI gain from pre-pregnancy to 15–20 weeks of gestation, history of GDM and thyroid diseases were risk factors for GDM. There were significant interactions between pre-pregnancy BMI and age (*P* < 0.05) after adjustment for other confounders. The odds ratio (OR) of pre-pregnancy BMI ≥ 30 kg/m^2^ at the age of <30 years, 30–34 years and ≥35 years was 1.30 (95% CI: 0.74–2.28, *P* = 0.36), 3.21 (95% CI: 2.28–4.52, *P* < 0.0001) and 1.55 (95% CI: 1.02–2.36, *P* = 0.0424), respectively. This indicated that pre-pregnancy BMI ≥ 30 kg/m^2^ had a stronger effect on GDM in the group aged 30–34 years than those under 30 years old.

**Conclusions:** The incidence of GDM was high in Qingdao. Overweight and obesity prior to pregnancy, gestational BMI gain from conception to 15–20 weeks of gestation and older age were correlated with an increased risk of GDM. Public health measures may be helpful to prevent excessive gestational weight gain.

## Introduction

Gestational diabetes mellitus (GDM) is associated with an increased risk of perinatal mortality and morbidity (1) and is a major public health concern. The prevalence of GDM has increased in recent decades in parallel with older age at conception and Westernized lifestyles, accompanied by an economic boom (2). Globally, GDM is estimated to affect 13.9% of all pregnancies (3). It is also associated with ischemic heart disease and type 2 diabetes (4, 5). The children of pregnant women with GDM are more likely to suffer from pediatric cardiovascular diseases and metabolic problems in later life (6). A meta-analysis suggested that the prevalence of GDM was 20.9% in Asia (7), and 14.8% in China (8). There has been an increase trend in the prevalence of GDM globally, including in China (9–11).

Obesity and maternal age are the two most important factors independently affecting the risk of GDM (12–15). With the end of the one-child policy in China, more women are getting pregnant at an older age, and probably a higher body mass index (BMI). It has been shown that ~17–21% of pregnant women were either overweight or obese, with an overall mean age at pregnancy of 30.7 years (16, 17). However, there is little recent data on incidence of GDM. Previous studies have demonstrated that BMI was correlated with increased risks of hypertension, stroke and cardiovascular disease (18–20), and that hypertension was associated with diabetes (21–23). GDM plays an important role in the pathogenesis of diabetes: about half of mothers with GDM will develop diabetes within 10 years, making GDM one of the strongest predictors of diabetes (5). However, the age differences in the association between obesity and GDM remain unclear. In this study, we hypothesized that the association between pre-pregnancy BMI and GDM varies with age.

The Women and Children's Health Care Study, conducted from August 2018, was a cohort study in Qingdao to explore recent GDM incidence and associated risk factors in pregnant women. Its objectives included: (1) to determine the current incidence of GDM; and (2) to evaluate the risk factors for GDM, such as the interaction between pre-pregnancy BMI and age. Our study provides an update on the assessment of age differences in the association between obesity and GDM, covering the entire population of pregnant women in Qingdao.

## Materials and Methods

### Data Collection

Qingdao is the third largest city in Shandong Province with a population of 9 million people and 10 districts. The Women and Children's Health Care Center System, which holds comprehensive data about regular health examinations, pre-pregnancy and delivery, was established in Qingdao in 2018. The central database for information is governed by Qingdao Women and Children's Hospital. Pregnant women use their ID number as the unique identification number linking information in different stages of gestation. Antenatal care is provided by the maternal and child health care system. This system includes 64 delivery hospitals, 65 prenatal screening blood collection hospitals, 10 district-level women and children's health centers and a municipal women and children's health care center (Qingdao Women and Children's Hospital). Qingdao Women and Children's Hospital is the coordination institution of the Women and Children's Health Care System. The 65 prenatal screening blood collection hospitals are the antenatal care providers [Down's screening-a test for prenatal detection of Trisomy 21 (Down's syndrome)] for pregnant women. This system includes detailed information about all pregnant women in Qingdao until their delivery.

The inclusion criteria were Qingdao household registration pregnant women within 15 to 20 weeks of gestation; aged 20 years and older. Exclusion criteria were: pregnant women with pre-existing diabetes, multiple pregnancies, difficulty with communication. Data on demographic information, folic acid supplements, parity, habitual use of tobacco, alcohol, and history of GDM were collected using standard and structured questionnaires. Relative literatures were reviewed to develop the questionnaire and to include possible variables that address the objective of the study. In the qualitative phase, the panel of experts in the field were invited to evaluate and discuss the essentiality of the questionnaire items, its wording, scaling, and its relevance. Before the actual data collection, the questionnaire was tested by asking 100 pregnant women.

Responses from participants were checked by trained interviewers to improve the validity of the self-reported data. Women were defined as having a smoking habit if they smoked at least one cigarette per day and kept smoking for at least 3 months before/during pregnancy. They were defined as having a habit of drinking if they ever drank alcohol before/during pregnancy. At the screening visit for GDM, the women were asked to fill in another questionnaire to record the results of an oral glucose tolerance test (OGTT).

### Definitions

The incidence rate was calculated as the total number of GDM in the cohort divided by sum of the pregnant women. GDM was diagnosed in accordance with the International Association of Diabetes and Pregnancy Study Group recommendation(IADPSG) for GDM based on 75 g 2-h OGTT: a fasting glucose ≥5.1 mmol/L (92 mg/dl), or a 1-h result ≥10.0 mmol/L (180 mg/dl), or a 2-h result ≥8.5 mmol/L (153 mg/dl) (14). Thyroid disease was diagnosed in line with the American Thyroid Association (ATA) recommendation: first trimester, thyroid stimulating hormone (TSH) levels between 0.1 and 2.5 mIU/L; second trimester, TSH between 0.2 and 3.0 mIU/L (24). Pre-pregnancy BMI was categorized according to the World Health Organization criteria. BMI <25 kg/m^2^ was considered as lean or healthy, BMI between 25 and 29 kg/m^2^ was considered as overweight, and BMI ≥ 30 kg/m^2^ was considered as obese. We collected the information on dietary patterns (well-balanced diet (eat both vegetables and meat), eat less vegetables, eat less meat) of pregnant women before pregnancy. Education level was categorized as low (received no education or primary school), medium (secondary school or high school) and high (college/university or above). The subjects reported their occupational physical activity levels following three categories: (1) light (mostly sitting for office work, e.g., secretary), (2) moderate (standing and walking, e.g., store assistant, light industrial worker), and (3) active (walking and lifting, heavy manual labor, e.g., industrial or farm worker) (25).

### Statistical Analysis

Continuous variables were described as mean ± SD and were compared by *t*-test; non-normal distributed continuous variables were expressed as median with interquartile range (IQR) and compared with the Mann-Whitney *U*-test; the categorical variables were expressed as numbers and percentages and were compared by the Chi-square test. The age-adjusted incidence of GDM was assessed by the Logistic regression analysis (26). GDM incidence trend tests across age varied by pre-pregnancy BMI were conducted Cochran-Armitage test. Multivariate logistic regression analysis was performed to evaluate the interaction between age (reference group, <30 years) and pre-pregnancy BMI (reference group, <25 kg/m^2^). If the interaction was of statistical significance, strata-specific analysis was then performed. Multivariate logistic regression analysis was used to detect any independent association between the risk factors and GDM. Two-sided tests with *P* < 0.05 were considered statistically significant. All analyses were performed by SAS software version 9.2 (SAS Institute Inc., Cary, NC, USA).

## Results

### Basic Characteristics of the Study Groups

We used data from August 1, 2018, to March 1, 2019. Of the 18,759 pregnant women, information on baseline characteristics was missing for 20, 1,563 did not receive an OGTT at 24–28 gestational weeks, and 31 had a history of diabetes. After excluding these 1,614 women (8.6%), a total of 17,145 participants were included in the analysis for this study. Written informed consent was obtained from all the participants and this study was approved by the Institutional Review Board of Qingdao Women and Children's Hospital Ethics.

Characteristics of women with and without GDM are shown in Table 1. Overall, the incidence and age-adjusted incidence of GDM were 17.42 and 17.45%. The mean age was 30.20 (SD: 4.62) years old, and the mean pre-pregnancy BMI was 22.43 (SD: 3.54) kg/m^2^; 2,980 women (17.38%) were overweight, and 611 (3.56%) were obese. In comparison with women without GDM, those with GDM were older (31.49 years vs. 29.93 years), and more likely to be obese (5.59 vs. 3.14%), have an active occupational physical activity (12.96 vs. 10.16%), have a parity of more than one (58.24 vs. 51.43%), have had assisted reproduction (2.08 vs. 1.10%), and have a greater gestational BMI gain from pre-pregnancy to 15–20 weeks' gestation (1.0 vs. 0.8 kg/m^2^). Pregnant women with GDM were more likely to have a history of GDM (17.09 vs. 2.65%). Women without GDM were also more likely to have a higher level of education (58.37 vs. 54.22%), eat less meat (11.90 vs. 8.58%) and have anemia (2.66 vs. 1.81%) (all *P* < 0.05).

**Table 1:** Characteristics of the study population according to gestational diabetes mellitus.

**Characteristics**	**Non-GDM (*N* = 14,159)**	**GDM (*N* = 2,986)**	**ALL**	***P*-value**
Urban,%	10,609 (74.93)	2,246 (75.22)	12,855 (74.98)	0.74
Age, mean (SD), year	29.93 ± 4.56	31.49 ± 4.72	30.20 ± 4.62	**<0.0001**
**Age group, year**				
<30	6,952 (49.10)	1,093 (36.60)	8,045 (46.92)	**<0.0001**
30-34	4,829 (34.11)	1,064 (35.63)	5,893 (34.37)	
≥35	2,378 (16.79)	829 (27.76)	3,207 (18.71)	
**Education level**				
Low	175 (1.24)	45 (1.51)	220 (1.28)	**0.0001**
Medium	5,719 (40.39)	1,322 (44.27)	7,041 (41.07)	
High	8,265 (58.37)	1,619 (54.22)	9,884 (57.65)	
**Occupational physical activity**				
Light	8,466 (59.79)	1,712 (57.33)	10,178 (59.36)	**<0.0001**
Moderate	4,254 (30.04)	887 (29.71)	5,141 (29.99)	
Active	1,439 (10.16)	387 (12.96)	1,826 (10.65)	
Pre-pregnancy BMI, mean (SD), kg/m^2^	22.22 ± 3.47	23.40 ± 3.70	22.43 ± 3.54	**<0.0001**
**Pre-pregnancy BMI group, kg/m**^**2**^				
<25	11,476 (81.05)	2,078 (69.59)	13,554 (79.06)	**0.0001**
25–30	2,239 (15.81)	741 (24.82)	2,980 (17.38)	
≥30	444 (3.14)	167 (5.59)	611 (3.56)	
**BMI at 15–20 weeks gestation, kg/m**^**2**^				
<25	10,117 (73.93)	1,730 (59.19)	11,847 (71.33)	**<0.0001**
25–30	2,930 (21.41)	938 (32.09)	3,868 (23.29)	
≥30	638 (4.66)	255 (8.72)	893 (5.38)	
BMI gain from pre-pregnancy to 15–20 weeks gestation	0.8 (0.1–1.6)	1.0 (0.3–1.8)	0.8 (0.2–1.6)	**<0.0001**
Parity≥1,%	7,282 (51.43)	1,739 (58.24)	9,021 (52.62)	**<0.0001**
Assisted reproduction,%	156 (1.10)	62 (2.08)	218 (1.27)	**<0.0001**
History of previous GDM,%	231 (2.65)	322 (17.09)	553 (5.22)	**<0.0001**
**Cigarette smokers, %**				
Habitual smoker before pregnancy	86 (0.61)	25 (0.84)	111 (0.65)	0.15
Habitual smoker during pregnancy	20 (0.14)	8 (0.27)	28 (0.16)	0.13
**Alcohol drinkers, %**				
Alcohol drinker before pregnancy	240 (1.70)	56 (1.88)	296 (1.73)	0.49
Alcohol drinker during pregnancy	31 (0.22)	10 (0.33)	41 (0.24)	0.24
Husband smoking before pregnancy,%	5,266 (37.19)	1,101 (36.87)	6,367 (37.14)	0.74
Husband drinking before pregnancy,%	4,604 (32.52)	1,006 (33.69)	5,610 (32.72)	0.21
**Dietary patterns**				
Well-balanced diet	12,074 (85.30)	2,648 (88.74)	14,722 (85.90)	**<0.0001**
Eat less vegetables	396 (2.80)	80 (2.68)	476 (2.78)	
Eat less meat	1,684 (11.90)	256 (8.58)	1,940 (11.32)	
Folic acid supplements,%	13,264 (93.68)	2,795 (93.60)	16,059 (93.67)	0.88
**Co-morbidities**				
Anemia	377 (2.66)	54 (1.81)	431 (2.51)	**0.0067**
Thyroid diseases	462 (3.26)	116 (3.88)	578 (3.37)	0.09

*Data are presented as means ± SD, median (IQR) or n (%). Variables with statistical significance were shown in boldface*.

### Incidence of GDM by Age and Residence/Pre-pregnancy BMI

**Figure 1:**
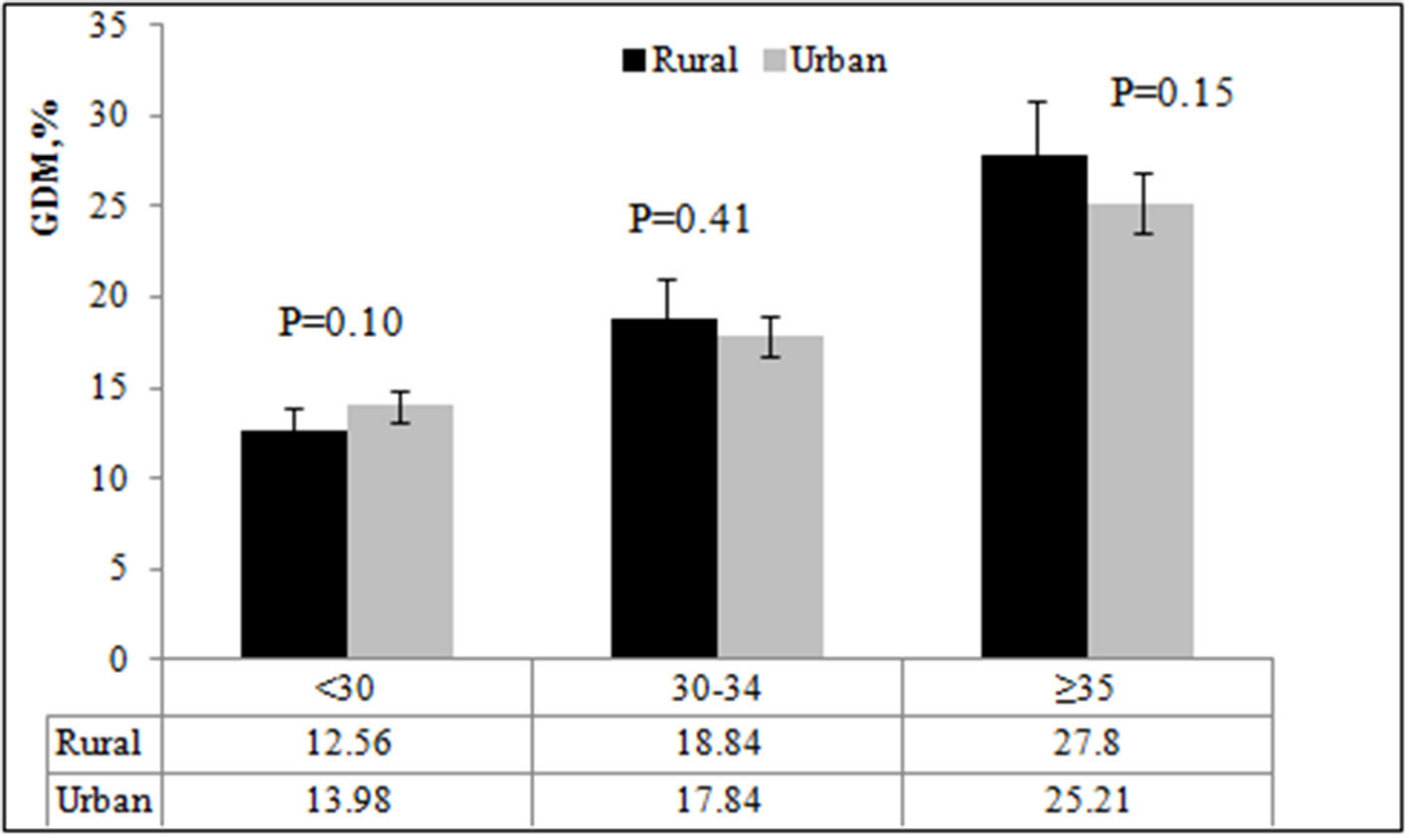
The incidence of GDM by age and place of residence.

Figure 1 shows the incidence of GDM by age in rural and urban areas. The incidence of GDM was similar in rural and urban areas (*P* = 0.74). The incidence of GDM in the three age groups was 13.98, 17.84, and 25.21%, respectively, in urban areas; and 12.56, 18.84, and 27.80%, respectively, in rural areas. There was no significant difference in the incidence of GDM among pregnant women in rural and urban areas (all *P* ≥ 0.05).

**Figure 2:**
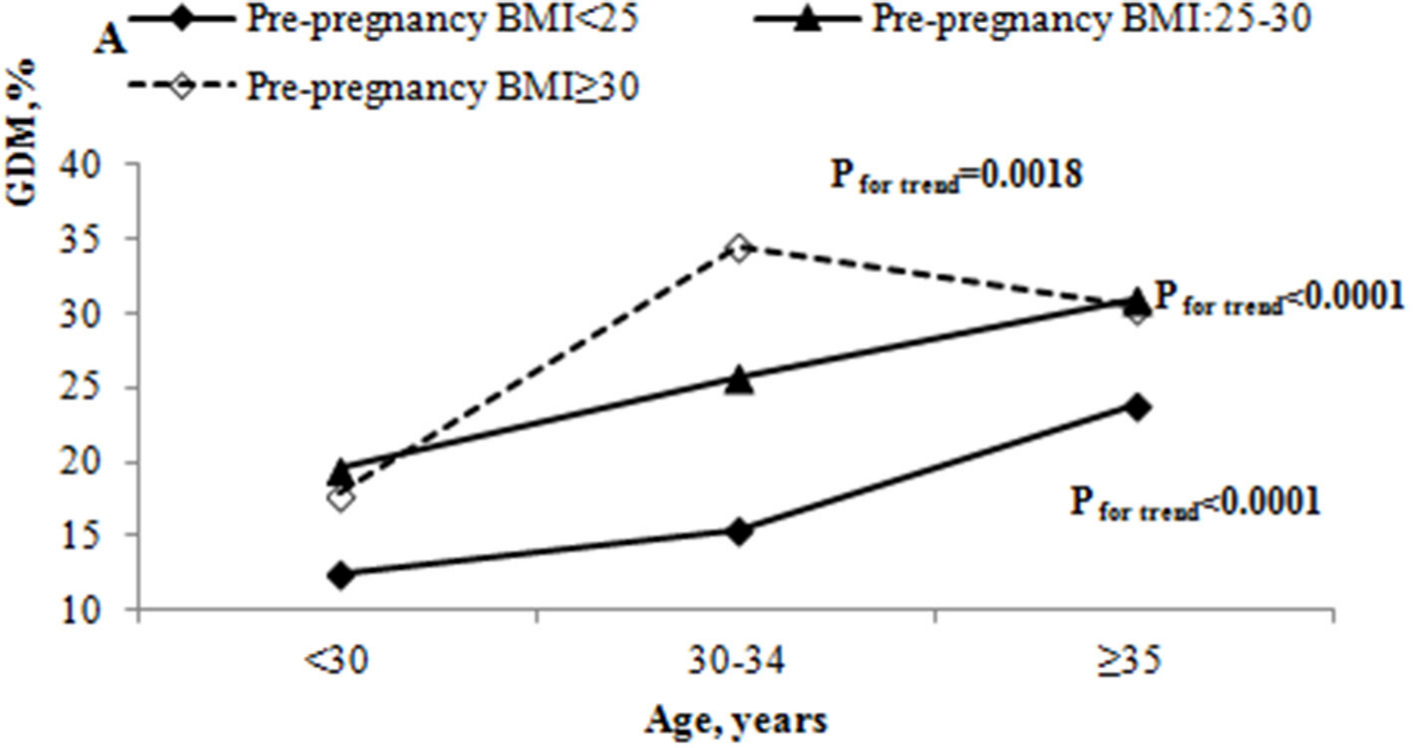
The incidence of GDM by age and pre-pregnancy BMI.

We also studied the incidence of GDM by age and pre-pregnancy BMI. The incidence of GDM by pre-pregnancy BMI were 12.46, 19.53 and 17.83% among those under 30 years; 15.37, 25.79, and 34.57% among those 30–34 years; and 23.75, 30.97, and 30.43% among those aged 35 years or older, respectively. The incidence of GDM appeared to increase steadily with age in all pre-pregnancy BMI groups (all *P* < 0.05) (Figure 2). The incidence of GDM was highest in the 30–34 years old group with pre-pregnancy BMI ≥ 30 kg/m^2^. Pregnant women with pre-pregnancy overweight or obese had a higher incidence of GDM among women aged 30 years or older when compared with pre-pregnancy <25 kg/m^2^ (all *P* < 0.05).

### Risk Factors for Gestational Diabetes Mellitus

Table 2 shows the results of multiple logistic regression analysis on associations between potential risk factors and GDM. After adjustment for potential risk factors, age ≥ 30 years, BMI gain from pre-pregnancy to 15–20 weeks of gestation, history of GDM and thyroid diseases were significantly associated with the risk of GDM (all *P* < 0.05).

**Table 2:** Factors associated with the incidence of gestational diabetes mellitus by multivariate logistic regression models.

**Factors**	**OR**	**95% CI**	***P*-value**
Residence: Urban (reference)	1.00		
Rural	0.99	0.87–1.13	0.91
Age groups: <30 (reference)	1.00		
30–34	1.24	1.05–1.46	**0.0116**
≥35	2.18	1.84–2.59	**<0.0001**
Education level: low (reference)	1.00		
Medium	0.86	0.58–1.26	0.43
High	0.76	0.52–1.14	0.18
Occupational physical activity:light (reference)	1.00		
moderate	0.93	0.82–1.05	0.24
active	1.09	0.92–1.30	0.30
Pre–pregnancy BMI group: <25 (reference)	1.00		
25–30	1.63	1.26–2.11	**0.0002**
≥30	1.30	0.75–2.26	0.34
BMI gain from pre–pregnancy to 15–20 weeks gestation	1.04	1.01–1.07	**0.0040**
Assisted reproduction:no (reference)	1.00		
Yes	1.51	0.93–2.45	0.10
Parity: 0 (reference)	1.00		
Parity≥1	1.06	0.91–1.24	0.47
GDM history: no (reference)	1.00		
Yes	7.74	6.42–9.32	**<0.0001**
Cigarette Smokers before or during pregnancy:no (reference)	1.00		
Yes	1.83	0.83–4.04	0.13
Alcohol drinkers before or during pregnancy:no (reference)	1.00		
Yes	0.94	0.62–1.44	0.79
Husband smoking before pregnancy: no (reference)	1.00		
Yes	1.01	0.90–1.13	0.87
Husband drinking before pregnancy: no (reference)	1.00		
Yes	1.10	0.98–1.23	0.12
Dietary patterns:Well–balanced diet (reference)	1.00		
Eat less vegetables	1.01	0.70–1.46	0.96
Eat less meat	0.82	0.67–1.00	0.05
Folic acid supplements: no (reference)	1.00		
Yes	1.03	0.84–1.26	0.76
Anemia: no (reference)	1.00		
Yes	0.91	0.65–1.29	0.60
Thyroid diseases: no (reference)	1.00		
Yes	1.47	1.11–1.96	**0.0073**
30–34^*^Overweight	1.18	0.86–1.63	0.30
30–34^*^Obesity	2.43	1.28–4.61	**0.0064**
≥35^*^Overweight	0.89	0.64–1.23	0.47
≥35^*^Obesity	1.20	0.61–2.39	0.60

*Variables with statistical significance were shown in boldface*.

* *The interactions between pre-pregnancy BMI and age*.

### The Effect of the Interaction Between Pre-pregnancy BMI and Age on GDM

**Figure 3:**
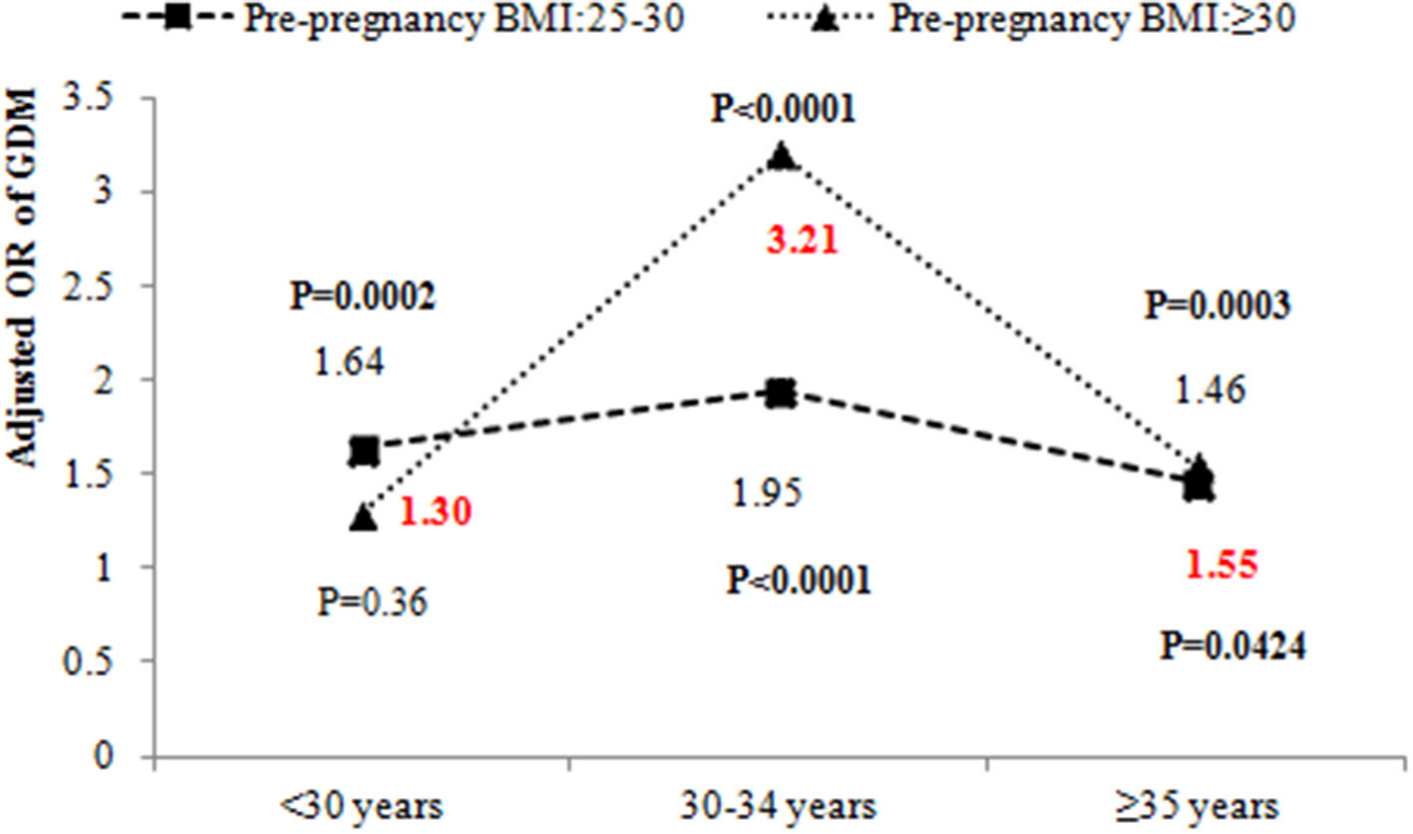
The odds ratio of pre-pregnancy overweight and obesity on GDM by different age groups. The adjusted ORs of GDM by age and pre-pregnancy BMI were adjusted for place of residence, education, occupational physical activity, BMI gain from pre-pregnancy to 15–20 weeks' gestation, assisted reproduction, dietary patterns, parity, GDM history, alcohol drinking before or during pregnancy, cigarette smoking before or during pregnancy, alcohol drinking of husband before pregnancy, cigarette smoking of husband before pregnancy, folic acid supplements, anemia, and thyroid diseases.

Table 2 also shows the results of testing the interactions between pre-pregnancy BMI and age. There was a significant interaction between pre-pregnancy BMI and age (*P* < 0.05) after adjustment for place of residence, education, occupational physical activity, BMI gain from pre-pregnancy to 15–20 weeks of gestation, assisted reproduction, dietary patterns, parity, GDM history, alcohol drinking before or during pregnancy, cigarette smoking before or during pregnancy, alcohol drinking of husband before pregnancy, cigarette smoking of husband before pregnancy, folic acid supplements, anemia and thyroid diseases. The odds ratio (OR) of pre-pregnancy BMI ≥ 30 kg/m^2^ and 30–34 years was 2.43 (95% CI: 1.28–4.61, *P* = 0.0064). We further evaluated the effect of pre-pregnancy BMI on GDM stratified by age (Figure 3). The OR of pre-pregnancy BMI ≥ 30 kg/m^2^ in women under 30 years old and 35 years and older was 1.30 (95% CI: 0.74–2.28, *P* = 0.36) and 1.55 (95% CI: 1.02–2.36, *P* = 0.0424). However, the OR of pre-pregnancy BMI ≥ 30 kg/m^2^ in the 30–34 years old group was 3.21 (95% CI: 2.28–4.52, *P* < 0.0001), reflecting a stronger effect of pre-pregnancy BMI ≥ 30 kg/m^2^ on the 30–34 year old group than women under 30 years old and 35 years and older groups.

## Discussion

In this large prenatal cohort study, we noted a high incidence of GDM. Overweight and obesity prior to pregnancy, gestational BMI gain from conception to 15–20 weeks of gestation and older age were correlated with an increased risk of GDM. We also observed a significant interaction between pre-pregnancy BMI and age. Having a pre-pregnancy BMI ≥ 30 kg/m^2^ had a stronger effect on GDM in the 30–34 years old than in those under 30 years old and 35 years and older groups.

The prevalence of GDM was reported as 20.9% in a meta-analysis of 84 studies (7), which was similar to our results (17.42%). Our incidence of GDM was higher than a previous analysis of a prospective population-based study of 18,589 pregnant women in Tianjin (9.3%) (27). Advancing maternal age is a known risk factor for GDM (28–30) and this discrepancy may be attributable to the differences in participants' ages (30.2 vs. 28.5 years). Our study also reported a 118% increased risk of GDM among women aged 35 years or older. This result shows that the incidence of GDM increased with age, and advancing maternal age may be the reason for the high incidence of GDM. Our study reinforced the findings of previous studies and indicated pregnancy is better planned before the age of 35 years, and that we should improve the GDM screening strategies for older pregnant women.

There are several studies confirming a strong association between obesity and the development of GDM (31–33). A study in the USA (34) found the overall population-attributable fraction was 46.2%, meaning nearly half of GDM events could have been avoided if these mothers had a normal pre-pregnancy BMI. Animal experiments have also shown that adipose tissue macrophages in obese mice secrete miRNA-containing exosomes, molecules that induce glucose intolerance and insulin resistance when administered to lean mice (35). However, we are not aware of any previous studies assessing the interactive effect of age and pre-pregnancy BMI on the development of GDM. Our study explicitly examined how age modified the effect of pre-pregnancy obesity on the development of GDM, and showed that this effect was particularly important in the 30–34 years old group. The results suggest that the underlying mechanisms causing GDM might differ by maternal age and indicated that weight reduction might be most beneficial to lower the risk of developing GDM in women aged 30–34 years. The variation by age in the association between obesity and GDM was a more complex issue. Stewart and colleagues showed that obesity in pregnancy could increase inflammatory status and that inflammation was related to advanced maternal age, which is an important risk factor for GDM (36, 37). Previous studies also showed that glucose tolerance impaired with age and that obesity was associated with insulin resistance and receptor abnormalities (38, 39). Development of insulin resistance with age may be a consequence of obesity. Older women with obesity may therefore be more prone to developing GDM. The mechanism for the association between pre-pregnancy obesity and GDM in those aged 30–34 years is unknown. It may be because pregnant women ≥35 years old were defined as having advanced maternal age (40). Childbearing women of advanced maternal age may be more likely to be healthy and exercise prudent health choices to reduce the effect of obesity on GDM (41). However, advanced maternal age continues to be associated with GDM and the association has changed little in the last few decades (42). More research is needed to establish the underlying mechanisms. Given no previous studies assessing the interactive effect of age and pre-pregnancy BMI on the development of GDM, we anticipate that our findings will be confirmed in further studies.

We observed a higher incidence of GDM in women with an excessive gestational BMI gain during the early stages of gestation. A cohort study in Norway showed the estimated risk for GDM increased with weight gain among both normal weight and overweight (43). A study in Beijing also showed BMI gain before 24 weeks was a risk factor for GDM (44). Weight management before conception and preventing excessive weight gain in the early stages of gestation may play a vital role in preventing GDM. Another important finding was thyroid diseases were related to an increased risk of GDM. Several studies showed an increased risk of insulin resistance with thyroid dysfunction, including hypothyroidism and hyperthyroidism (45, 46), which was in accordance with our findings. Thyroid hormone played a critical role in the control of insulin secretion and glucose homeostasis (47). Pregnancy was related to critical and complicated changes in maternal thyroid hormone, failure to adapt the physiological changes would lead to thyroid dysfunction (48). This study demonstrated an independent effect of thyroid disease on GDM and thyroid disease may be offered predicted the occurrence of GDM in early pregnancy.

Our study found anemia was associated with GDM. However, the association did not persist after the adjusting for relevant confounders. Lao and colleagues conducted a retrospective case-control study and showed anemia was confirmed to be significantly associated with decreased prevalence of GDM (49). Pregnant women with GDM with an increased ferritin concentration and it was therefore logical to hypothesize that pregnant women with anemia would have a reduced likelihood of GDM (50). The decreased prevalence of GDM in pregnant women with anemia may be explained by the combined effects of iron deficiency (49). Excessive iron affected the synthesis and secretion of insulin, and enhances lipid oxidation, thereby reducing glucose utilization in muscles and increasing gluconeogenesis in the liver, leading to liver-mediated insulin resistance (51). It indicated the routine iron prescription for non-anemic pregnant women should be reappraised.

The strengths of this study lie in its large prospective cohort, which enabled the entire population of pregnant women in Qingdao to be investigated, without selection bias. This gave a high-quality study design and we also had a high response rate. Interviewers were trained to improve the validity of the self-reported data. However, our study did have some limitations. First, height and weight before conception was provided by the pregnant women, which may have resulted in recall bias. Furthermore, we did not measure the blood pressure of all pregnant women, so we cannot evaluate the effect of pregnancy-induced hypertension on GDM in this study. We also cannot exclude residual confounders related to lifestyle factors. However, we found an interaction between age and pre-pregnancy BMI, which may have important public health implications. We hope our study may provide a new perspective on GDM in China and facilitate further research to improve public health.

## Conclusion

In conclusion, GDM diagnosed on basis of the IADPSG criteria was common among pregnant women in Qingdao. Overweight and obesity before conception, gestational BMI gain from pre-pregnancy to 15–20 weeks' gestation and older age were associated with an increased risk of GDM. A pre-pregnancy BMI ≥ 30 kg/m^2^ had a stronger effect on GDM among those age 30–34 years. This suggests that, pregnancy is better planned before the age of 35 years and weight reduction might be more beneficial for women aged 30–34 years to lower the risk of developing GDM. Public health measures targeted at weight management before and during pregnancy may have an impact on future preventive strategies for GDM.

## Data Availability Statement

The raw data supporting the conclusions of this article will be made available by the authors, without undue reservation.

## Ethics Statement

The studies involving human participants were reviewed and approved by Institutional Review Board of Qingdao Women and Children's Hospital Ethics. The patients/participants provided their written informed consent to participate in this study.

## Author Contributions

QX, YX, GL, TW, and WN: conducted the literature review and analyses and drafted the manuscript, and approved the final manuscript as submitted. AZ and JZ: conducted the data collection and analysis, and critically reviewed the manuscript, and approved the final manuscript to be published. All authors contributed to the article and approved the submitted version.

## Conflict of Interest

The authors declare that the research was conducted in the absence of any commercial or financial relationships that could be construed as a potential conflict of interest.

## References

[1] Jovanovic L Pettitt DJ. Gestational diabetes mellitus. JAMA. (2001) 286:2516–8. 10.1001/jama.286.20.2516 11722247

[2] Simjak P Cinkajzlova A Anderlova K Parizek A Mraz M Krsek M. The role of obesity and adipose tissue dysfunction in gestational diabetes mellitus. J Endocrinol. (2018) 238:R63–R77. 10.1530/JOE-18-0032 29743342

[3] Hod M Kapur A Sacks DA Hadar E Agarwal M Di Renzo GC The International Federation of Gynecology and Obstetrics (FIGO) Initiative on gestational diabetes mellitus: a pragmatic guide for diagnosis, management, and care. Int J Gynaecol Obstetr. (2015) 131 (Suppl. 3):S173–211. 10.1016/S0020-7292(15)30033-3 26433807

[4] Daly B Toulis KA Thomas N Gokhale K Martin J Webber J. Increased risk of ischemic heart disease, hypertension, and type 2 diabetes in women with previous gestational diabetes mellitus, a target group in general practice for preventive interventions: a population-based cohort study. PLoS Med. (2018) 15:e1002488. 10.1371/journal.pmed.1002488 29337985 PMC5770032

[5] Damm P Houshmand-Oeregaard A Kelstrup L Lauenborg J Mathiesen ER Clausen TD. Gestational diabetes mellitus and long-term consequences for mother and offspring: a view from Denmark. Diabetologia. (2016) 59:1396–99. 10.1007/s00125-016-3985-5 27174368

[6] Leybovitz-Haleluya N Wainstock T Landau D Sheiner E. Maternal gestational diabetes mellitus and the risk of subsequent pediatric cardiovascular diseases of the offspring: a population-based cohort study with up to 18 years of follow up. Acta Diabetol. (2018) 55:1037–42. 10.1007/s00592-018-1176-1 29936651

[7] Lee KW Ching SM Ramachandran V Yee A Hoo FK Chia YC Prevalence and risk factors of gestational diabetes mellitus in Asia: a systematic review and meta-analysis. BMC Preg Childbirth. (2018) 18:494 10.1186/s12884-018-2131-4 PMC6295048 30547769

[8] Gao C Sun X Lu L Liu F Yuan J. The prevalence of gestational diabetes mellitus in mainland China: a systematic review and meta-analysis. J Diabetes Investig. (2019) 10:154–62. 10.1111/jdi.12854 29683557 PMC6319492

[9] Zhang F Dong L Zhang CP Li B Wen J Gao W. Increasing prevalence of gestational diabetes mellitus in Chinese women from 1999 to 2008. Diab Med. (2011) 28:652–7. 10.1111/j.1464-5491.2010.03205.x 21569085

[10] Wang Y Chen L Xiao K Horswell R Besse J Johnson J. Increasing incidence of gestational diabetes mellitus in Louisiana, 1997-2009. J Women's Health. (2012) 21:319–25. 10.1089/jwh.2011.2838 22023415

[11] Rajab KE Issa AA Hasan ZA Rajab E Jaradat AA. Incidence of gestational diabetes mellitus in Bahrain from 2002 to 2010. Int J Gynaecol Obstetr. (2012) 117:74–7. 10.1016/j.ijgo.2011.11.013 22265190

[12] Solomon CG Willett WC Carey VJ Rich-Edwards J Hunter DJ Colditz GA. A prospective study of pregravid determinants of gestational diabetes mellitus. JAMA. (1997) 278:1078–83. 10.1001/jama.278.13.1078 9315766

[13] Ferreira LAP Piccinato CA Cordioli E Zlotnik E. Pregestational body mass index, weight gain during pregnancy and perinatal outcome: a retrospective descriptive study. Einstein. (2019) 18:eAO4851. 10.31744/einstein_journal/2020AO4851 31721895 PMC6896599

[14] Black MH Sacks DA Xiang AH Lawrence JM. The relative contribution of prepregnancy overweight and obesity, gestational weight gain, and IADPSG-defined gestational diabetes mellitus to fetal overgrowth. Diabetes Care. (2013) 36:56–62. 10.2337/dc12-0741 22891256 PMC3526206

[15] Liang Y Li DT Chen MX Gong YH Zhang X Yang WY. [Associations of pre-pregnancy body mass index and gestational weight gain with gestational diabetes mellitus: a cohort study in southwest China]. Sichuan da xue xue bao Yi xue ban. (2019) 50:83–87. 31037910

[16] Chen YH Li L Chen W Liu ZB Ma L Gao XX. Pre-pregnancy underweight and obesity are positively associated with small-for-gestational-age infants in a Chinese population. Scient Rep. (2019) 9:15544. 10.1038/s41598-019-52018-7 31664141 PMC6820714

[17] Zhuang C Gao J Liu J Wang X He J Sun J. Risk factors and potential protective factors of pregnancy-induced hypertension in China: a cross-sectional study. J Clin Hypert. (2019) 21:618–23. 10.1111/jch.13541 30990249 PMC8030480

[18] †LABMP Dongfeng Gu MD Whelton MR Xiqui Wu MD Chung-Shiuan Chen MS Xiufang Duan MD. Body mass index and risk of stroke among Chinese men and women. Ann Neurol. (2010) 67:11. 10.1002/ana.21950 20186847 PMC4371851

[19] Rodgers A Pan WH Mhurchu CN Gu DF Woodward M. Body mass index and cardiovascular disease in the Asia-Pacific Region: an overview of 33 cohorts involving 310 000 participants. Int J Epidemiol. (2004) 33:751. 10.1093/ije/dyh163 15105409

[20] Humayun A Shah AS Sultana R. Relation of hypertension with body mass index and age in male and female population of Peshawar, Pakistan. J Ayub Med Coll Abbottabad. (2009) 21:63–65. 20929016

[21] Wilson PW Meigs JB Sullivan L Fox CS Nathan DM D'Agostino RB Sr. Prediction of incident diabetes mellitus in middle-aged adults: the Framingham Offspring Study. Arch Internal Med. (2007) 167:1068–74. 10.1001/archinte.167.10.1068 17533210

[22] Gress TW Nieto FJ Shahar E Wofford MR Brancati FL. Hypertension and antihypertensive therapy as risk factors for type 2 diabetes mellitus. Atherosclerosis Risk in Communities Study. N Engl J Med. (2000) 342:905–12. 10.1056/NEJM200003303421301 10738048

[23] Golden SH Wang NY Klag MJ Meoni LA Brancati FL. Blood pressure in young adulthood and the risk of type 2 diabetes in middle age. Diabetes Care. (2003) 26:1110–5. 10.2337/diacare.26.4.1110 12663582

[24] Stagnaro-Green A Abalovich M Alexander E Azizi F Mestman J Negro R. Guidelines of the American Thyroid Association for the diagnosis and management of thyroid disease during pregnancy and postpartum. Thyroid. (2011) 21:1081–125. 10.1089/thy.2011.0087 21787128 PMC3472679

[25] Hu G Eriksson J Barengo NC Lakka TA Valle TT Nissinen A. Occupational, commuting, and leisure-time physical activity in relation to total and cardiovascular mortality among Finnish subjects with type 2 diabetes. Circulation. (2004) 110:666–73. 10.1161/01.CIR.0000138102.23783.94 15277321

[26] Roalfe AK Holder RL Wilson S. Standardisation of rates using logistic regression: a comparison with the direct method. BMC Health Serv Res. (2008) 8:275. 10.1186/1472-6963-8-275 19113996 PMC2661894

[27] Junhong L Ping S Cuiping Z Huiguang T Fuxia Z Shuang Z. Prevalence of gestational diabetes mellitus and its risk factors in Chinese pregnant women: a prospective population-based study in Tianjin, China. PLoS ONE. (2015) 10:e0121029. 10.1371/journal.pone.0121029 25799433 PMC4370728

[28] Lin J Fu Y Han Q Yan J Chen R Zhang H. Gestational weight management and pregnancy outcomes among women of advanced maternal age. Exp Ther Med. (2019) 18:1723–28. 10.3892/etm.2019.7752 31410130 PMC6676176

[29] Makgoba M Savvidou MD Steer PJ. An analysis of the interrelationship between maternal age, body mass index and racial origin in the development of gestational diabetes mellitus. BJOG. (2012) 119:276–82. 10.1111/j.1471-0528.2011.03156.x 22044452

[30] Yang H Wei Y Gao X Xu X Fan L He J. Risk factors for gestational diabetes mellitus in Chinese women: a prospective study of 16,286 pregnant women in China. Diabet Med. (2009) 26:1099–104. 10.1111/j.1464-5491.2009.02845.x 19929987

[31] Leddy MA Power ML Schulkin J. The impact of maternal obesity on maternal and fetal health. Rev Obstet Gynecol. (2008) 1:170–8. 19173021 PMC2621047

[32] Janevic T Zeitlin J Egorova N Balbierz A Howell EA. The role of obesity in the risk of gestational diabetes among immigrant and U.S.-born women in New York City. Ann Epidemiol. (2018) 28:242–48. 10.1016/j.annepidem.2018.02.006 29501220 PMC5875722

[33] Groof Z Garashi G Husain H Owayed S AlBader S Mouhsen H. Prevalence, risk factors, and fetomaternal outcomes of gestational diabetes mellitus in kuwait: a cross-sectional study. J Diabetes Res. (2019) 2019:9136250. 10.1155/2019/9136250 30944829 PMC6421795

[34] Kim SY England L Wilson HG Bish C Satten GA Dietz P. Percentage of gestational diabetes mellitus attributable to overweight and obesity. Am J Public Health. (2010) 100:1047–52. 10.2105/AJPH.2009.172890 20395581 PMC2866592

[35] Ying W Riopel M Bandyopadhyay G Dong Y Birmingham A Seo JB. Adipose tissue macrophage-derived exosomal mirnas can modulate in vivo and in vitro insulin sensitivity. Cell. (2017) 171:372–84 e12. 10.1016/j.cell.2017.08.035 28942920

[36] Stewart FM Freeman DJ Ramsay JE Greer IA Caslake M Ferrell WR. Longitudinal assessment of maternal endothelial function and markers of inflammation and placental function throughout pregnancy in lean and obese mothers. J Clin Endocrinol Metab. (2007) 92:969–75. 10.1210/jc.2006-2083 17192290

[37] Khambule L George JA. The role of inflammation in the development of GDM and the use of markers of inflammation in GDM screening. Adv Exp Med Biol. (2019) 1134:217–42. 10.1007/978-3-030-12668-1_12 30919340

[38] Helseth R Salvesen O Stafne SN Morkved S Salvesen KA Carlsen SM. Gestational diabetes mellitus among Nordic Caucasian women: prevalence and risk factors according to WHO and simplified IADPSG criteria. Scand J Clin Lab Invest. (2014) 74:620–8. 10.3109/00365513.2014.928942 24980704

[39] Tovar A Must A Bermudez OI Hyatt RR Chasan-Taber L. The impact of gestational weight gain and diet on abnormal glucose tolerance during pregnancy in Hispanic women. Matern Child Health J. (2009) 13:520–30. 10.1007/s10995-008-0381-x 18597166 PMC2683196

[40] Resta RG. Changing demographics of advanced maternal age (AMA) and the impact on the predicted incidence of Down syndrome in the United States: implications for prenatal screening and genetic counseling. Am J Med Genet A. (2005) 133A:31–6. 10.1002/ajmg.a.30553 15637725

[41] Viau PA Padula CA Eddy B. An exploration of health concerns & health-promotion behaviors in pregnant women over age 35. MCN Am J Matern Child Nurs. (2002) 27:328–34. 10.1097/00005721-200211000-00006 12439134

[42] Carolan M Frankowska D. Advanced maternal age and adverse perinatal outcome: a review of the evidence. Midwifery. (2011) 27:793–801. 10.1016/j.midw.2010.07.006 20888095

[43] Sorbye LM Skjaerven R Klungsoyr K Morken NH. Gestational diabetes mellitus and interpregnancy weight change: a population-based cohort study. PLoS Med. (2017) 14:e1002367. 10.1371/journal.pmed.1002367 28763446 PMC5538633

[44] Zhu WW Yang HX Wang C Su RN Feng H Kapur A. High prevalence of gestational diabetes mellitus in beijing: effect of maternal birth weight and other risk factors. Chin Med J (Engl). (2017) 130:1019–25. 10.4103/0366-6999.204930 28469095 PMC5421170

[45] Leng J Li W Wang L Zhang S Liu H Li W. Higher thyroid-stimulating hormone levels in the first trimester are associated with gestational diabetes in a Chinese population. Diabet Med. (2019) 36:1679–85. 10.1111/dme.14106 31407386

[46] Chaker L Ligthart S Korevaar TI Hofman A Franco OH Peeters RP. Thyroid function and risk of type 2 diabetes: a population-based prospective cohort study. BMC Med. (2016) 14:150. 10.1186/s12916-016-0693-4 27686165 PMC5043536

[47] Sarah Crunkhorn MEP. Links between thyroid hormone action, oxidative metabolism, and diabetes risk? Thyr Offic J Am Thyr Assoc. (2008) 18:227. 10.1089/thy.2007.0249 18279023

[48] Männistö T Vääräsmäki M Pouta A Hartikainen AL Suvanto E. Thyroid dysfunction and autoantibodies during pregnancy as predictive factors of pregnancy complications and maternal morbidity in later life. J Clin Endocrinol Metab. (2010) 95:1084. 10.1210/jc.2009-1904 20080846

[49] Lao TT Ho LF. Impact of iron deficiency anemia on prevalence of gestational diabetes mellitus. Diabetes Care. (2004) 27:650–6. 10.2337/diacare.27.3.650 14988280

[50] Lao TT Chan PL Tam KF. Gestational diabetes mellitus in the last trimester - a feature of maternal iron excess? Diab Med A J Br Diab Assoc. (2010) 18:218–23. 10.1046/j.1464-5491.2001.00453.x 11318843

[51] DeFronzo A. R. The triumvirate:β-cell, muscle, liver-A collusion responsible for NIDDM. Diabetes. (1988) 37:667–87. 10.2337/diab.37.6.667 3289989

